# Long-Term Exposure to Sucrose during the Differentiation Process Increases Permeability Independently of TAS1R3 in In Vitro Models of the Intestinal Barrier Function

**DOI:** 10.1021/acs.jafc.5c02797

**Published:** 2025-07-16

**Authors:** Markus L. Rechl, Evelin Balika, Sascha Oberle, Verena Preinfalk, Sarah Stadlmayr, Jana Rasztovits, Jakob P. Ley, Barbara Lieder

**Affiliations:** † Christian Doppler Laboratory for Taste Research, Faculty of Chemistry, 27258 University of Vienna, Josef-Holaubek-Platz 2, Vienna 1090, Austria; ‡ Vienna Doctoral School in Chemistry (DoSChem), Währinger Straße 42, Vienna 1090, Austria; § Institute of Physiological Chemistry, Faculty of Chemistry, University of Vienna, Josef-Holaubek-Platz 2, Vienna 1090, Austria; ∥ 10784 Symrise AG, Muehlenfeldstrasse 1, Holzminden 37603, Germany; ⊥ Institute of Clinical Nutrition, Department of Human Nutrition and Dietetics, University of Hohenheim, Fruwirthstr. 12, Stuttgart 70593, Germany

**Keywords:** sweet taste receptor, monoculture, coculture, tight junctions, permeability, noncaloric sweeteners

## Abstract

While some studies report negative effects of noncaloric sweeteners and high glucose on models for the intestinal barrier function, the long-term effects of noncaloric sweeteners and sucrose remain unclear. Here, we investigated the impact of a long-term treatment with caloric and noncaloric sweeteners on two in vitro models for the intestinal barrier function. A Caco-2 monoculture and a coculture with mucus-producing HT29-MTX-E12 cells were treated with equi-sweet and equi-molar concentrations of sucrose, sucralose, rebaudioside M, and neohesperidin dihydrochalcone during the differentiation period of 21 days. Only treatment with 150 mM sucrose increased the paracellular permeability by up to 259% in the monoculture independent of the sweet taste receptor subunit TAS1R3 and osmotic pressure. Sucrose treatment decreased the gene expression of pore-forming markers while increasing sealing tight junctions without increasing tight junction protein 1 on the protein level. The coculture with HT29-MTX-E12 cells was more resistant toward the treatments and osmotic stress than the Caco-2 monoculture, suggesting a beneficial effect of the produced mucus by the HT29-MTX-E12 cells. In conclusion, treatment with sucrose showed a time-dependent effect on markers of intestinal permeability independent of TAS1R3 and osmotic pressure, with a potential protective effect of the mucus layer.

## Graphical abstract

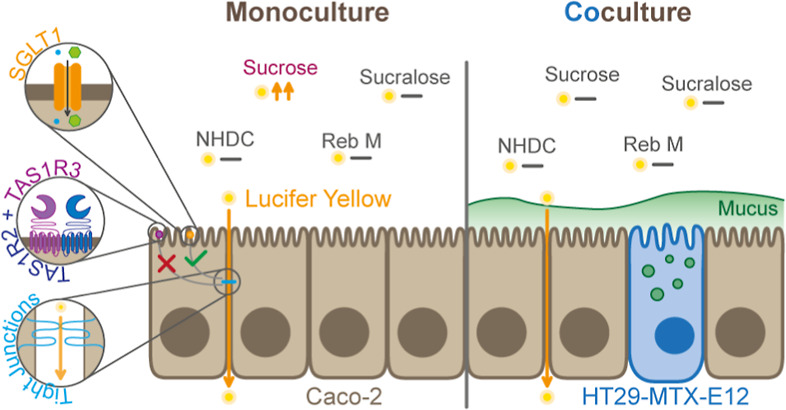

## 1 Introduction

The intestinal barrier represents a frontier between the internal milieu and the environment, protecting the organism from pathogens and antigens. In addition to its protective function, the intestinal barrier plays an important role in the absorption of electrolytes, water, and nutrients.^1^ The barrier function is mainly regulated by tight junction (TJ) proteins, which control paracellular permeability. A large variety of TJ proteins have been identified over the years; however, the interaction of the TJs is still not fully understood. Some members are referred to as sealing TJs and support the formation of impermeable barriers, while others form pores that are permeable for small molecules, such as water and ions. Important representatives for the sealing TJs are the tight junction protein 1, occludin, claudin-1, and claudin-4. In contrast to the sealing TJ, the addition of claudin-2 cDNA to Madin–Darby canine kidney (MDCK) I cells reduced the barrier function of claudin-1/4-based TJ strands.^2^ A disruption of this important barrier can lead to an increased permeability.

Food ingredients can also contribute to the opening of TJs and thus increase the paracellular permeability. The contribution of the monosaccharide glucose has been intensively investigated in this context.^3–5^ The glucose-initiated opening of the paracellular space starts with the activation of sodium-glucose cotransporter 1 (SGLT1), which subsequently activates the myosin light chain kinase (MLCK). This is followed by the contraction of the actomyosin ring, resulting in an opening of TJs and thus increasing the paracellular permeability.^1,3,6^ Beside the activation of the SGLT1, inflammation has also been shown to activate MLCK.^7^ As a further mechanism for increased paracellular permeability, a pathway involving the sweet taste receptor subunit TAS1R3 is discussed, based on short-term studies using the noncaloric sweeteners sucralose and aspartame on undifferentiated Caco-2 cells.^8^ In more detail, concentrations of 0.1 mM were shown to increase the paracellular permeability after short-term exposure in this study. According to the authors, these concentrations are physiologically achievable,^8^ depending on dietary choices and given that one can of soft drink has concentrations up to 0.5 mM sucralose (68 mg/12 oz).^9^

While previous studies focused mostly on short-term effects of glucose or noncaloric sweet tasting compounds, the consequences of long-term treatments and direct comparisons are not well investigated. A long-term study demonstrated that the usage of high glucose medium (25 mM), compared to normal physiological glucose concentration (5.5 mM), increased the permeability of FITC-dextran and altered the abundance of TJ proteins in Caco-2 and HT29-MTX cells.^4^

In addition, the impact of sucrose on the intestinal barrier function is not well investigated. Thus, in this study, we compared the impact of the caloric sweetener sucrose on markers of the intestinal barrier function with the noncaloric sweeteners sucralose, neohesperidin dihydrochalcone (NHDC), and rebaudioside M in equi-sweet and equi-molar concentrations in two in vitro models. For that purpose, we selected the well-established Caco-2 cell line. In order to establish models that represent the physiological function more closely, cocultures of Caco-2 cells and mucus-producing goblet cells like HT29-MTX-E12 cells^10^ have been used.^4,11^ While the usage of coculture models is closer to physiological conditions,^12^ the direct comparison to monocultures is often missing, leaving the question of comparability to previous studies unanswered. Therefore, we compared the effects of the Caco-2 monoculture to a coculture model of Caco-2 and HT29-MTX-E12 cells.

## 2 Materials and Methods

### 2.1 Materials

The test compounds rebaudioside M (96%) and sucralose (>99%) were kindly provided by Symrise AG (Holzminden, Germany). Sucrose (≥99.5%) and neohesperidin dihydrochalcone (NHDC) (≥96%) were purchased from Sigma-Aldrich (Vienna, Austria), and d­(−)-mannitol (≥98%) was purchased from Carl Roth (Karlsruhe, Germany). Lactisole sodium salt (>98%) from Cayman Chemical was purchased from Biomol (Hamburg, Germany).

All other chemicals were obtained from Sigma-Aldrich (Vienna, Austria) unless indicated otherwise.

### 2.2 Cell Culture

For the cultivation of the cells, a growth medium consisting of Gibco DMEM, high glucose, GlutaMAX Supplement (Thermo Fisher Scientific, Vienna, Austria) with 10% fetal bovine serum (Gibco, Thermo Fisher Scientific, Vienna, Austria), and 1% Penicillin–Streptomycin was used. Caco-2 cells (CLS, Eppelheim, Germany) and HT29-MTX-E12 cells (Sigma-Aldrich, Vienna, Austria) were cultivated separately in a humidified incubator at 37 °C and 5% CO_2_. The cells were passaged at a confluency of 80–95%, and the medium was changed every 2–3 days. For all further experiments, a Caco-2 monoculture and a coculture of 90% Caco-2 cells and 10% HT29-MTX-E12 cells were used. The authenticity of the used cell lines was confirmed by the cell line authentication service from Microsynth AG (Balgach, Switzerland).

The long-term incubation with the different treatments started on day 2 after seeding and was maintained during the differentiation period. Sampling time points were days 7, 14, and 21 after seeding. The sweet tasting compounds sucrose, sucralose, NHDC, and rebaudioside M were used in an equi-molar (0.1 mM) and an equi-sweet concentration (equi-sweet to a 5% sucrose solution according to Karl et al.^13^). The equi-sweet concentrations were: 150 mM for sucrose, 0.2 mM for sucralose and rebaudioside M, and 0.1 mM for NHDC. For the noncaloric sweeteners, a 1000× stock in dimethyl sulfoxide (DMSO) was used, and a DMSO control (growth medium + 0.1% DMSO) was used for normalization of these treatments. To rule out an osmotic effect of sucrose (150 mM), mannitol was used as an osmotic control at the same concentration. For the inhibition of the sweet taste receptor subunit TAS1R3,^14^ 1 mM lactisole was used in combination with 150 mM sucrose.

### 2.3 Cell Viability

To exclude the negative effects of the treatments on the cell viability, a neutral red uptake assay was carried out according to the protocol of Repetto et al.^15^ on day 21 after seeding. Therefore, 6 × 10^4^ cells per well were seeded in 96-well plates (Sarstedt, Wiener Neudorf, Austria) and treated with the test compounds. On day 21, the cells were incubated in a humidified incubator (37 °C, 5% CO_2_) for 3 h with a 40 μg/mL neutral red solution in DMEM. After aspiration of the neutral red solution and a washing step with PBS, a destain solution (50% ethanol 96%, 49% deionized water, 1% glacial acetic acid) was added to extract the uptaken neutral red from the cells. After 10 min of shaking on a microplate shaker, the absorption was measured at 540 nm with a plate reader (Spark Tecan, Tecan, Grödig, Austria). The cell viability after treatment was calculated relative to the control, and a viability above 85% was considered sufficient to be used in further experiments.

### 2.4 Lucifer Yellow Assay

To determine the effect of the long-term treatment on the paracellular permeability, cells were seeded in translucent 24-well inserts (Sarstedt, Wiener Neudorf, Austria) with a 0.4 μm pore size and 0.3 cm^2^ growth area at a density of 1.5 × 10^5^ cells per well. The treatment of the monoculture and the coculture started on day 2 after seeding on the apical chamber. Permeability of lucifer yellow (LY) (Invitrogen, Thermo Fisher Scientific, Vienna, Austria) from the apical to the basal compartment was assessed on days 7, 14, and 21. The last medium change was carried out 2 h before the experiment, and the transepithelial electrical resistance (TEER) was measured with a chopstick electrode set for EVOM STX2 (World Precision Instruments, Sarasota, Florida) to confirm an intact monolayer. Two washing steps with 20 mM HEPES buffered Hanks’ Balanced Salt Solution (HBSS/HEPES) on the apical and basal sides were carried out. The basal chamber was filled with 1.2 mL fresh HBSS/HEPES and the apical chamber was filled with 0.5 mL 100 μM LY solution in HBSS/HEPES. The cells were incubated at 37 °C and a 0.5 mL sample from the basal chamber was taken after 15, 30, and 60 min. The removed volume in the basal chamber was replaced with fresh HBSS/HEPES after every sampling. The fluorescence (excitation: 428 nm, emission: 536 nm) of the samples was measured in duplicate with a FlexStation 3 (Molecular Devices, Munich, Germany). The total amount of lucifer yellow on the basal side was calculated and is presented relative to the corresponding control.

### 2.5 Gene Expression Analysis

For gene expression analysis with quantitative real-time PCR (qPCR), cells for the monoculture and the coculture were seeded at a density of 7.3 × 10^5^ cells per well in 12-well plates (Sarstedt, Wiener Neudorf, Austria). The mRNA was isolated using the Monarch Total RNA Miniprep Kit (New England Biolabs, Frankfurt am Main, Germany) according to the manufacturer’s protocol. The quality and quantity of the obtained RNA were evaluated spectrophotometrically with a NanoQuant Plate (Tecan, Austria) on a plate reader (Spark Tecan, Tecan, Grödig, Austria). Subsequently 1 μg of the isolated RNA was converted to cDNA with the LunaScript RT SuperMix Kit (New England Biolabs, Frankfurt am Main, Germany) following the manufacturer’s instructions. For the qPCR, Luna Universal qPCR Master Mix (New England Biolabs, Frankfurt am Main, Germany) was used. A list of the sequences of the primer pairs used during the qPCR is provided in Table 1 for genes encoding tight junction protein 1 (*TJP1*), claudin 1 (*CLDN1*), claudin 2 (*CLDN2*), claudin 4 (*CLDN4*), claudin 7 (*CLDN7*), occludin (*OCLN*), myosin light chain kinase (*MYLK*), sodium glucose cotransporter 1 (*SLC5A1*), hypoxanthine phosphoribosyltransferase 1 (*HPRT1*), and glyceraldehyde-3-phosphate dehydrogenase (*GAPDH*) and in Table S1 for the taste receptor type 1 member 3 (*TAS1R3*) and glucose transporter 2 (*SLC2A2*). New primer pairs designed with Primer BLAST (NCBI) or taken from PrimerBank^16–18^ were validated by sequencing the obtained PCR product (Eurofins Genomics, Vienna, Austria). Four biological samples (*n*) were measured in three technical replicates (tr) using real-time qPCR StepOnePlus (Applied Biosystems, Thermo Fisher, Vienna, Austria) or Bioer LineGene 9600 Plus (Hangzhou Bioer Technology, Hangzhou, China).

**Table 1:** Sequence of Forward (FW) and Reverse (RV) Primers of Reference Genes (*HPRT1* and *GAPDH*) and Genes of Interest

gene	sequence 5′–3′	*C*_final_ [nM]	source
*HPRT1*	FW: CCTGGCGTCGTGATTAGTGA	100	^31^
	RV: CGAGCAAGACGTTCAGTCCT		
*GAPDH*	FW: AGGTCGGAGTCAACGGATTTG	200	^32^
	RV: GGGGTCATTGATGGCAACAATA		
*TJP1*	FW: ACCAGTAAGTCGTCCTGATCC	100	^16–18^ ID: 116875766c2
	RV: TCGGCCAAATCTTCTCACTCC		
*CLDN1*	FW: CCAGTCAATGCCAGGTACGAA	100	^33^
	RV: CACACGTAGTCTTTCCCGCT		
*CLDN2*	FW: CGGGACTTCTACTCACCACTG	100	^33^
	RV: GGATGATTCCAGCTATCAGGGA		
*CLDN4*	FW: ATGGTGATAGTGCCGGTGTC	100	Primer-BLAST, NCBI
	RV: GCGGAGTAAGGCTTGTCTGT		
*CLDN7*	FW: CTGCAAAATGTACGACTCGGTG	100	^20^
	RV: GCAAGACCTGCCACGATGAAAA		
*OCLN*	FW: GTCTAGGACGCAGCAGATTG	100	^33^
	RV: CTGGCTGAGAGAGCATTGGT		
*MYLK*	FW: CCCGAGGTTGTCTGGTTCAAA	100	^16–18^ ID: 116008189c1
	RV: GCAGGTGTACTTGGCATCGT		
*SLC5A1*	FW: CCGATATCTCCATCATCGTTATCTAC	100	^32^
	RV: CACGATTGGTGGAAAACATAGC		

Theoretical start concentrations (*N*0-values) of the mRNA were calculated using LinRegPCR (version 2021.1).^19^ After normalization to the geometrical mean of the reference genes *HPRT1* and *GAPDH*, the fold change compared to the corresponding control was calculated.

### 2.6 Localization of TJP1

For the immunostaining of TJP1, a modified protocol from JanssenDuijghuijsen et al.^20^ was used. Cells were seeded on 1.5H coverslips (Marienfeld, Carl Roth, Karlsruhe, Germany) at a density of 3.64 × 10^5^ cells per well in 24-well plates (Sarstedt, Wiener Neudorf, Austria). On days 7, 14, and 21 after seeding, cells were fixed with 3.6% formaldehyde for 10 min at room temperature (RT). After the first washing step with PBS, a washing solution of 100 mM glycine in PBS was used to inactivate excess formaldehyde, followed by two additional washing steps with PBS. The fixed cells were permeabilized with 0.1% Triton-X100 in PBS for 20 min at RT and washed with PBS three times for 5 min each. Blocking was performed for 30 min at RT in PBS supplemented with 2% BSA (Carl Roth, Karlsruhe, Germany), 0.2% horse-serum (Invitrogen, Thermo Fisher Scientific), and 0.1% Triton-X100 (Carl Roth, Karlsruhe, Germany). To visualize TJP1, the cells were incubated with the primary rabbit antibody against TJP1 (40-2200, Invitrogen, Thermo Fisher Scientific, Vienna, Austria) in a 1:100 dilution for 90 min. To visualize the bound primary antibodies, cells were incubated for 90 min with the secondary antibody Alexa Fluor 488-labeled goat anti-rabbit IgG (H + L) (A11008, Invitrogen, Thermo Fisher Scientific, Vienna, Austria) in a 1:200 dilution. After the primary and the secondary antibody incubation, three washing steps with PBS for 5 min each were carried out to remove unbound antibodies. For the staining of the nucleus, cells were incubated with Hoechst 33342 (Thermo Fisher Scientific, Vienna, Austria) in a 1:500 dilution for 10 min at RT. After three washing steps with PBS and two washing steps with ddH_2_O, the stained samples were transferred on a drop of anti-fade fluorescence Mounting Medium (Abcam, Cambridge, UK) on a glass slide.

The fluorescence of the samples was examined by confocal laser scanning microscopy (LSM 800, Carl Zeiss, Jena, Germany), with a 20× magnification objective (plan-apochromat 20*×*/0.8 M27, Carl Zeiss, Jena, Germany). For each biological sample, *z*-stacks with 12 layers with a spacing of 1 μm were acquired at three locations. To quantitate TJP1, each *z*-stack was merged, using the maximum intensity projection of Fiji/ImageJ (version 2.14.0/1.54f). A negative control without the primary antibody was used to confirm the specific binding of the secondary antibody. These images were used to determine the threshold for further analysis. Therefore, the mean and SD of the maximum intensity of three replicates was determined, and the threshold was set to 16,000 arbitrary units, based on the mean + 10× SD. For the samples, the mean intensity of the signals above this threshold of each image was analyzed and used for further statistical analysis.

### 2.7 Statistics

Data were analyzed using GraphPad Prism version 10.4.0 and R version 4.3.2.^21^ For robust ANOVA (trimmed on means), the package WRS2 version 1.1-5 ^22^ was used.

Data were checked for normal distribution (D’Agostino-Pearson omnibus K2) and homoscedasticity (Spearman’s test). In case the criteria for the ordinary one-way ANOVA were not fulfilled, the Kruskal–Wallis test with Dunn’s multiple comparison test was used to find significant differences (*p* < 0.05) between three or more groups. To conduct two- or three-way ANOVA, the robust model, based on trimmed means, was used. Differences between two groups were analyzed using the Mann–Whitney test.

## 3 Results

### 3.1 Characterization of the Cell Culture Models

As a first step, TEER values, permeability of LY and mucus abundance of Caco-2 and HT29-MTX-E12 cells, and a 9 + 1 coculture were measured. A time-dependent increase of TEER values could be observed for all three models (Figure S1A). The highest TEER values were found for the Caco-2 monoculture at all time points, followed by the coculture with HT29-MTX-E12 cells, while the lowest values were reached by the HT29-MTX-E12 monoculture.

Accordingly, the monoculture of HT29-MTX-E12 cells had the highest permeability for lucifer yellow on day 7 (7.2 ± 1.6%), which decreased over cultivation time (Figure S1B) to 4.5 ± 1.5% on day 14 and 2.9 ± 1.0% on day 21. In comparison, the coculture and the Caco-2 monoculture showed both a lower permeability of lucifer yellow compared to the HT29-MTX-E12 monoculture on days 7 and 14. The permeability of LY after 60 min was determined in the monoculture with 3.0 ± 0.5%, 2.8 ± 0.6%, and 2.6 ± 0.3% and the coculture with 2.6 ± 0.6%, 3.0 ± 0.5%, and 3.2 ± 0.4% for days 7, 14, and 21, respectively.

An Alcian blue staining (Figure S2) qualitatively confirmed an increased mucus abundance in the coculture after the addition of HT29-MTX-E12 cells.

Gene expression analysis (Figure S3) confirmed the expression of genes encoding the sweet taste receptor subunit TAS1R3 and the glucose transporters SGLT1 and GLUT2 in the Caco-2 monoculture and coculture with HT29-MTX-E12 cells. The highest expression levels were found for *SLC5A1* at all time points and for both models compared to the undifferentiated cells (day 0).

### 3.2 Cell Viability after Long-Term Treatment

The viability of cells after long-term treatment with the test compounds was assessed using neutral red uptake assays. None of the applied treatments reduced the viability below 85% of the respective control (Figure S4).

### 3.3 Sucrose Increases Paracellular Permeability Stronger in the Monoculture Model

To ensure the integrity of the monolayer, the TEER values were measured before the permeability assay and are presented in Figure S5. Next, we assessed the permeability of LY after long-term treatment with sucrose and noncaloric sweeteners in equi-sweet and equi-molar concentrations. The results of the monoculture and the coculture are depicted in Figure 1 and show an increased paracellular permeability of LY after treatment with sucrose in the equi-sweet concentration at all time points in the monoculture. The highest increase was measured on day 7 with a permeability of 259 ± 87% compared to the control. The effect of the sucrose treatment decreased over time to 224 ± 46% on day 14 and 186 ± 40% on day 21. Although the paracellular permeability after 150 mM sucrose treatment of the coculture was not significantly increased, the highest effect was reached on day 7 with 149 ± 32%, compared to the control. On days 14 and 21, the permeability of LY after 150 mM sucrose treatment was measured with 111 ± 30% and 141 ± 50%, respectively. In contrast to the equi-sweet sucrose, noncaloric sweeteners did not increase the permeability of LY compared to the control. In the equi-molar concentrations, no difference in permeability between treatments and control was detected.

**Figure 1:**
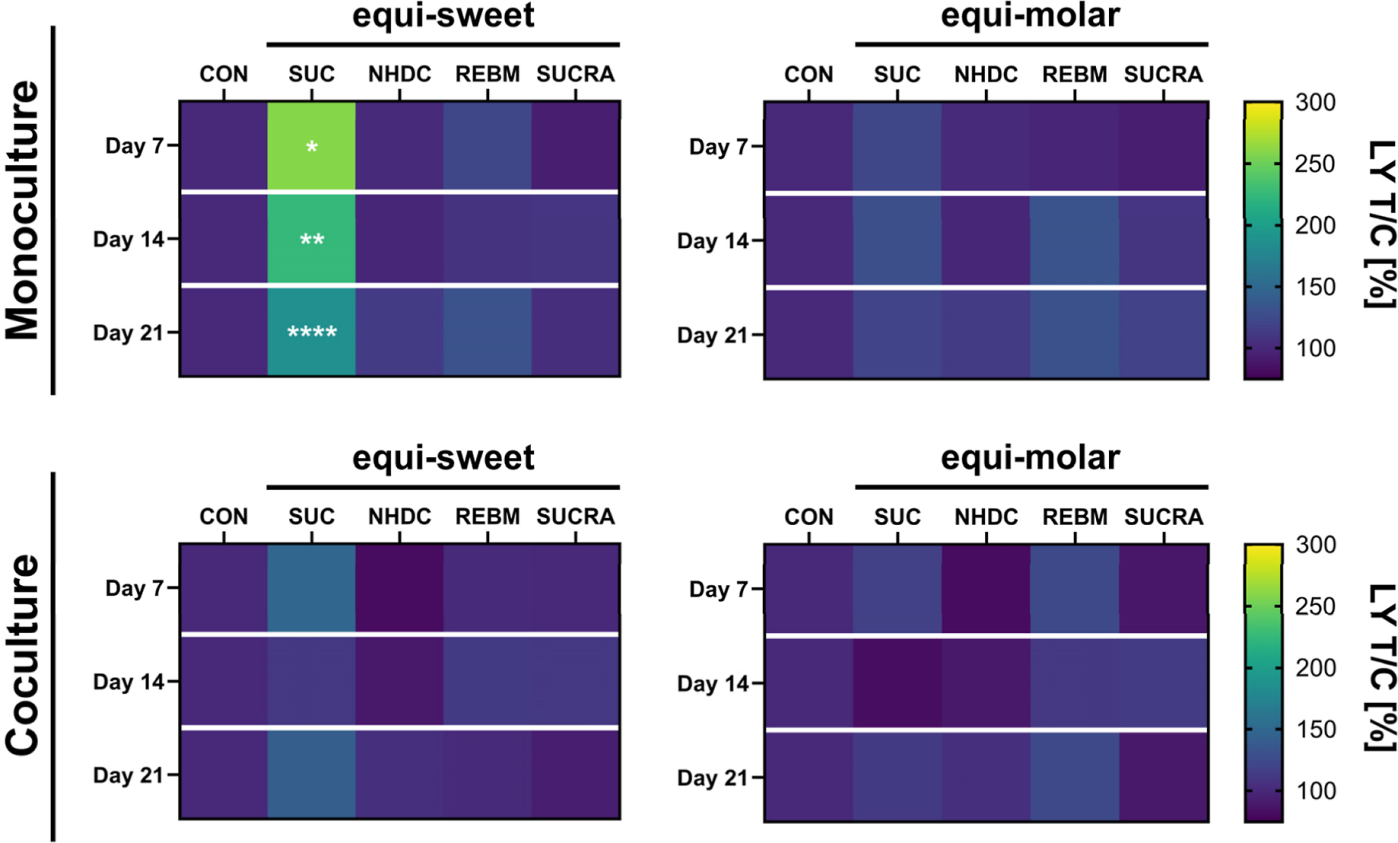
Paracellular permeability of lucifer yellow to the basal side after 60 min relative to the control in [%] for the Caco-2 monoculture and the coculture with HT29-MTX-E12 cells on days 7, 14, and 21 with the equi-sweet treatments 150 mM sucrose (SUC), 0.1 mM neohesperidin dihydrochalcone (NHDC), 0.2 mM rebaudioside M (REBM), and 0.2 mM sucralose (SUCRA) or in equi-molar concentration (0.1 mM). Data are presented as mean (*n* = 3–5, tr = 2). Statistical differences to the control (CON) in the corresponding week were determined using a Kruskal–Wallis test with Dunn’s multiple comparison test and is indicated with *p < 0.05, **p < 0.01, and ****p < 0.0001.

A robust three-way ANOVA on trimmed means revealed an impact of the model, treatment, and a combination of the model and treatment.

Based on this result, further analyses using 150 mM sucrose were conducted to explore the underlying mechanisms of the increased permeability after a high sucrose treatment.

### 3.4 The Sweet Taste Receptor TAS1R3 Is Not Involved in the Increased Permeability after Sucrose Treatment

The comparison of noncaloric sweeteners and sucrose in equi-sweet concentrations did not hint toward an involvement of the sweet taste receptor. However, since short-term treatments with certain sweeteners led to increased permeability in undifferentiated Caco-2 cells via activation of TAS1R3,^8^ we aimed to exclude a pathway involving TAS1R3 for sucrose. Thus, the sweet taste receptor subunit TAS1R3 inhibitor lactisole was applied in combination with 150 mM sucrose. The results of the monoculture and coculture after the addition of lactisole to the medium containing 150 mM sucrose are shown in Figure 2. In both models, the addition of lactisole did not decrease the permeability compared to the treatment with sucrose solely. In contrast, a small increased permeability could be detected on day 7 in the Caco-2 monoculture after the treatment with 1 mM lactisole in combination with 150 mM sucrose. Treatment with lactisole alone did not impact the permeability (Figure S6).

**Figure 2:**
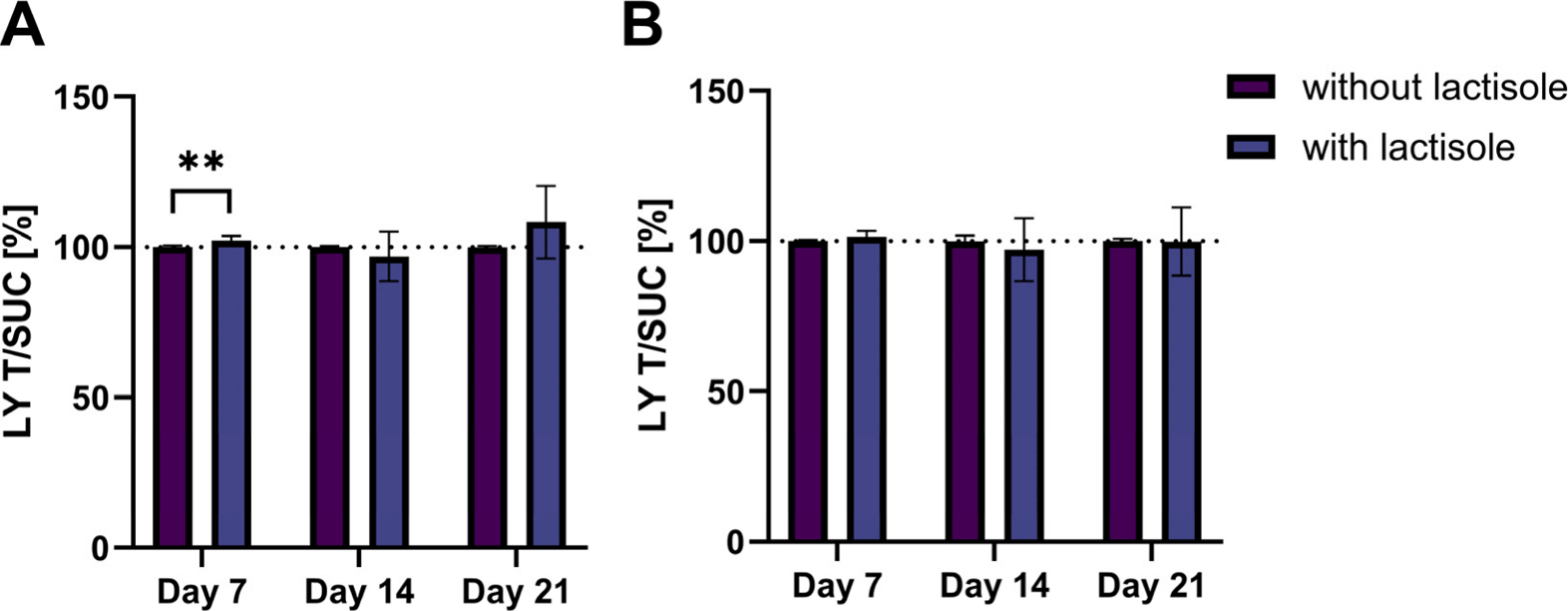
Paracellular permeability of lucifer yellow (LY) after treatment with 150 mM sucrose with or without the addition of 1 mM lactisole for the (A) monoculture and the (B) coculture on days 7, 14, and 21, normalized to the 150 mM sucrose treatment. Data presented as mean ± SD (*n* = 4, tr = 2–3). A significant difference of the two treatments was tested using the Mann–Whitney test and a significant increase is indicated with **p < 0.01.

### 3.5 Osmotic Pressure Is Not the Driving Force of the Increased Permeability after 150 mM Sucrose Treatment

To exclude osmotic pressure from the 150 mM sucrose treatment as the main driving force for the increased permeability, 150 mM mannitol was used as the osmotic control (Figure 3).

**Figure 3:**
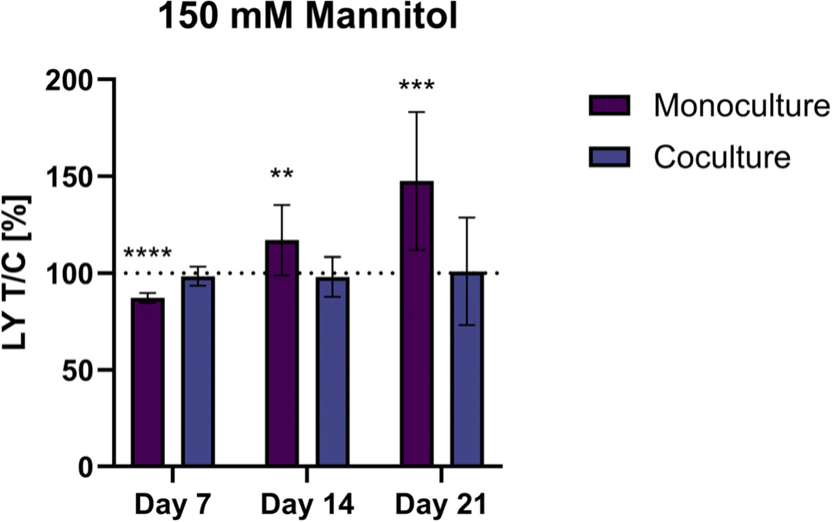
Paracellular permeability of lucifer yellow after treatment with 150 mM mannitol for the monoculture and coculture at days 7, 14, and 21, normalized to the control (100%). Data presented as mean ± SD (*n* = 5–7, tr = 2). A significant difference to the control was tested using the Mann–Whitney test and is indicated with **p < 0.01, ***p < 0.001, and ****p < 0.0001.

The monoculture and the coculture model showed different sensitivity toward this treatment. While the permeability of LY after osmotic stress with 150 mM mannitol stayed at the level of the control in the coculture model, the permeability of the monoculture model increased with time. In the monoculture, the permeability was decreased on day 7 to 87 ± 3%, followed by an increase to 117 ± 18% on day 14 and 147 ± 36% on day 21. This trend is contrary to the effect after sucrose treatment, where the permeability decreased with time. Although a significant increase after mannitol treatment could be found in the monoculture, the differences of mannitol with the control are moderate and smaller compared to the effect of the corresponding sucrose treatment.

### 3.6 Sucrose Treatment Decreases *SLC5A1* Gene Expression

The gene expressions of *SLC5A1*, encoding sodium glucose cotransporter 1 (SGLT1), and *MYLK*, encoding myosin light chain kinase, were analyzed after treatment with 150 mM sucrose or 150 mM mannitol.

A clear difference between the two treatments in both models could be observed for *SLC5A1* (Figure 4A,B). While *SLC5A1* expression was decreased after treatment with 150 mM sucrose at all time points, mannitol treatment increased the expression over time, compared to the control. Both treatments led to an upregulated gene expression of *MYLK* (Figure 4C,D) at nearly all time points in both cultures. However, the effect was higher after the sucrose treatment in the monoculture and the coculture compared to the mannitol treatment.

**Figure 4:**
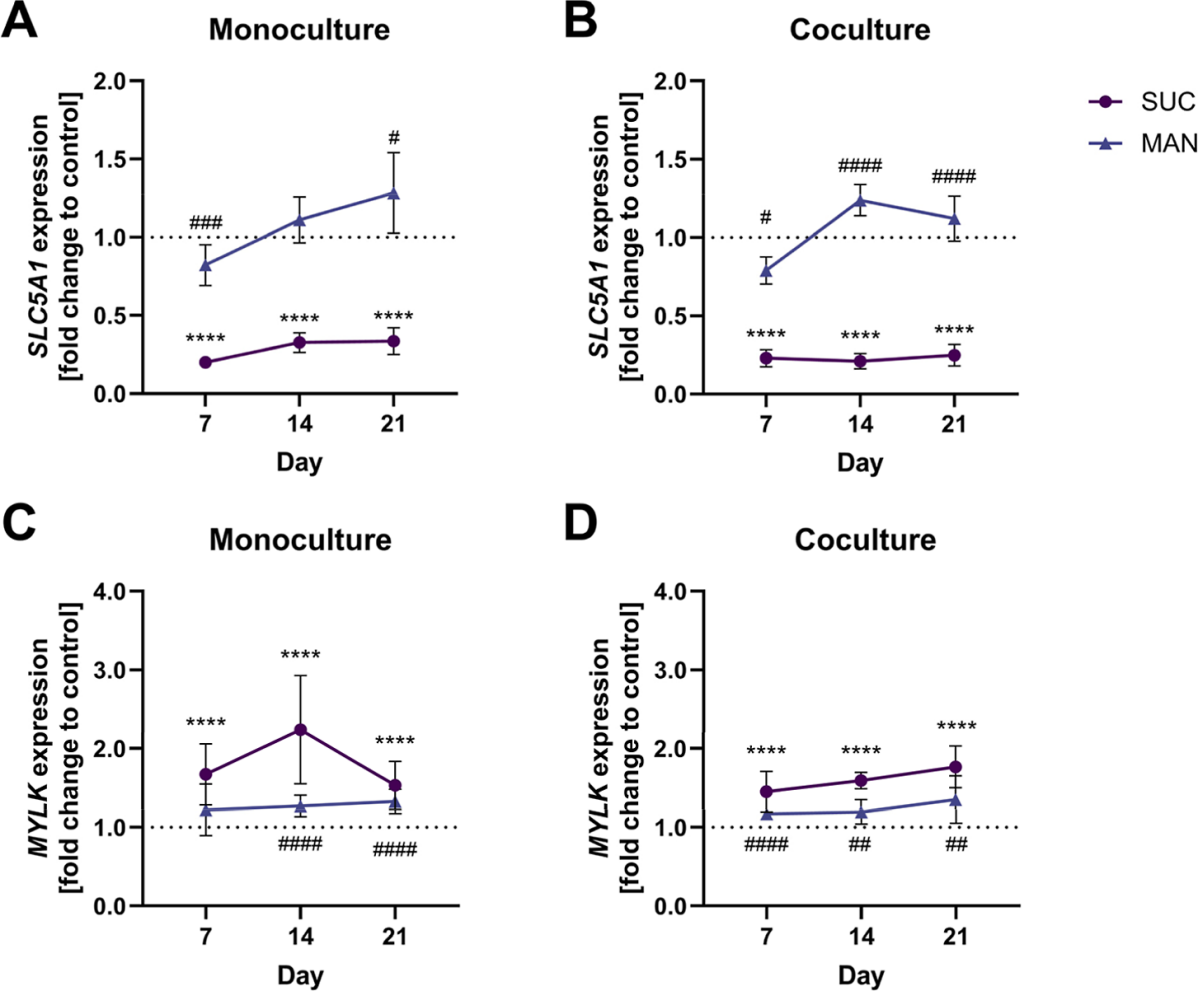
Gene expression analysis of *SLC5A1 and MYLK* for the (A,C) monoculture and the (B,D) coculture after 150 mM sucrose or 150 mM mannitol treatment on days 7, 14, and 21. Data are presented as mean ± SD (*n* = 4, tr = 2–3) of the fold change to the corresponding control. Statistical significance to the control was determined with the Mann–Whitney test and is indicated with *p < 0.05, **p < 0.01, ***p < 0.001, and ****p < 0.0001 for sucrose. In the same way, # is used for significant differences of the mannitol treatment to the control.

### 3.7 Gene Expression of Sealing Tight Junctions Is Upregulated through Sucrose and Mannitol Treatment

The gene expression of selected TJ genes (*TJP1, OCLN, CLDN1, CLDN2, CLDN4*, and *CLDN7*) was analyzed with qPCR and is depicted in Figure 5. The Caco-2 monoculture and the coculture with HT29-MTX-E12 cells share a similar pattern, regarding the trends and effects. Notably, the expression of genes encoding TJs that are important for the sealing of the paracellular space were upregulated at all time points after sucrose and mannitol treatment, indicating a counter regulation. Among all the TJ genes examined, *TJP1* had the highest upregulation over the entire period. The highest increase in *TJP1* expression was found after sucrose treatment on day 14 in the monoculture (2.13 ± 0.25) and on day 7 in the coculture (2.09 ± 0.24). Treatment with mannitol increased the gene expression for *TJP1* to a lower extent than sucrose treatment. In contrast to the high upregulation of the sealing TJs, the gene expression of the pore forming claudin-2 (*CLDN2*) was downregulated at all time points after sucrose treatment, indicating a counter regulation.

**Figure 5:**
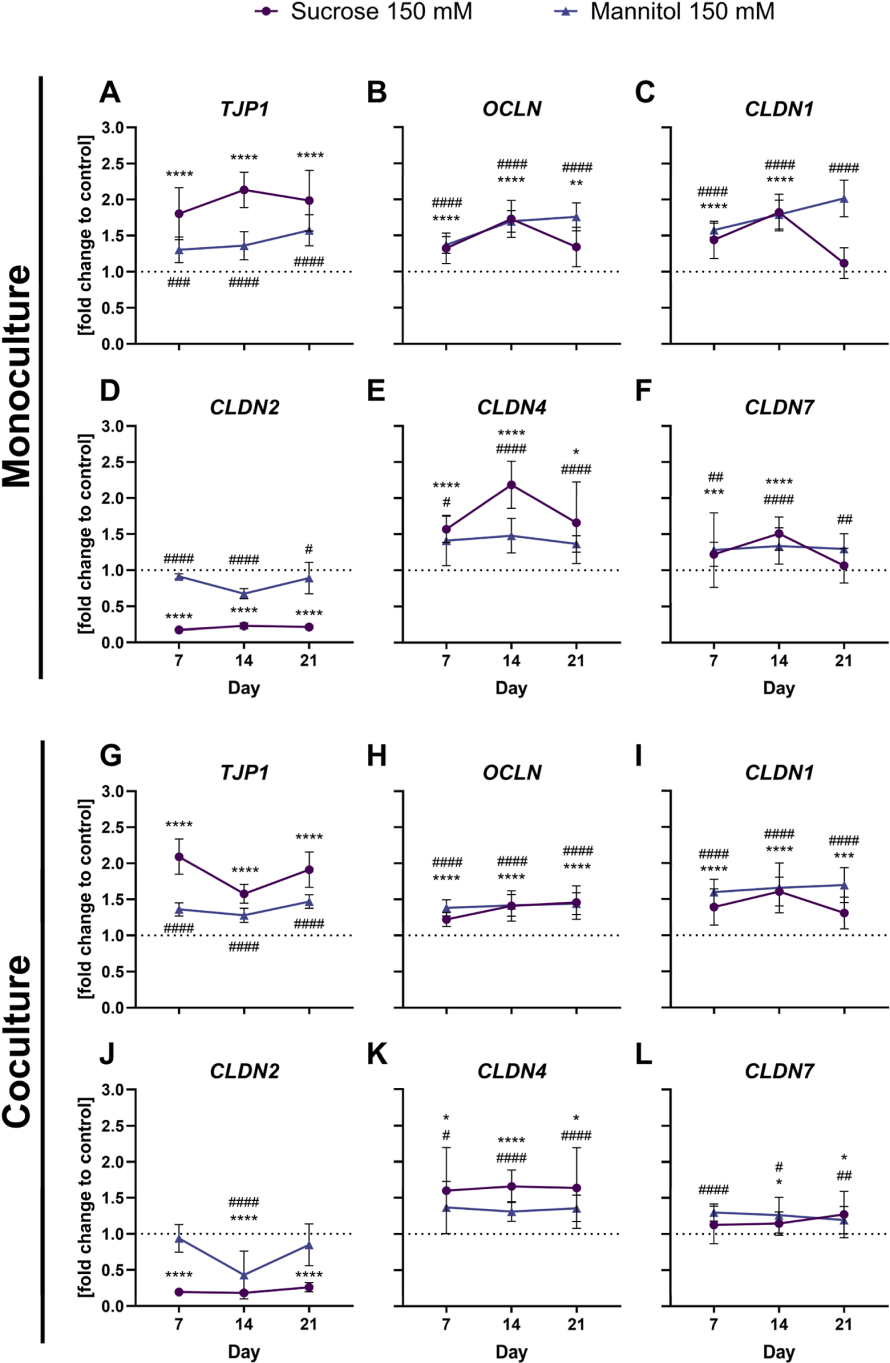
Gene expression of selected tight junction genes of the monoculture (A–F) and coculture (G–L) on days 7, 14, and 21 after treatment with 150 mM sucrose or mannitol. Data presented as mean ± SD (*n* = 4, tr = 2–3) of the fold change to the corresponding control. Statistical significance to the corresponding control was determined with the Mann–Whitney test and is indicated with *p < 0.05, **p < 0.01, ***p < 0.001, and ****p < 0.0001 for sucrose. In the same way, # is used for significant differences of the mannitol treatment to the control.

### 3.8 Sucrose Treatment Reduces TJP1 Abundance

The immunostaining of the TJP1 in Figure 6A,B showed a typical distribution of the protein at the cell boundaries for both models. As shown in Figure 6C,D, the fluorescence intensity of the control and the treatment groups increased from day 7 to day 14, after which a decrease was observed until day 21. TJP1 abundance was not influenced by the treatments on days 7 and 14 in both models. Only in the monoculture, a decreased intensity was found on day 21 after treatment with sucrose (Figure 6C).

**Figure 6:**
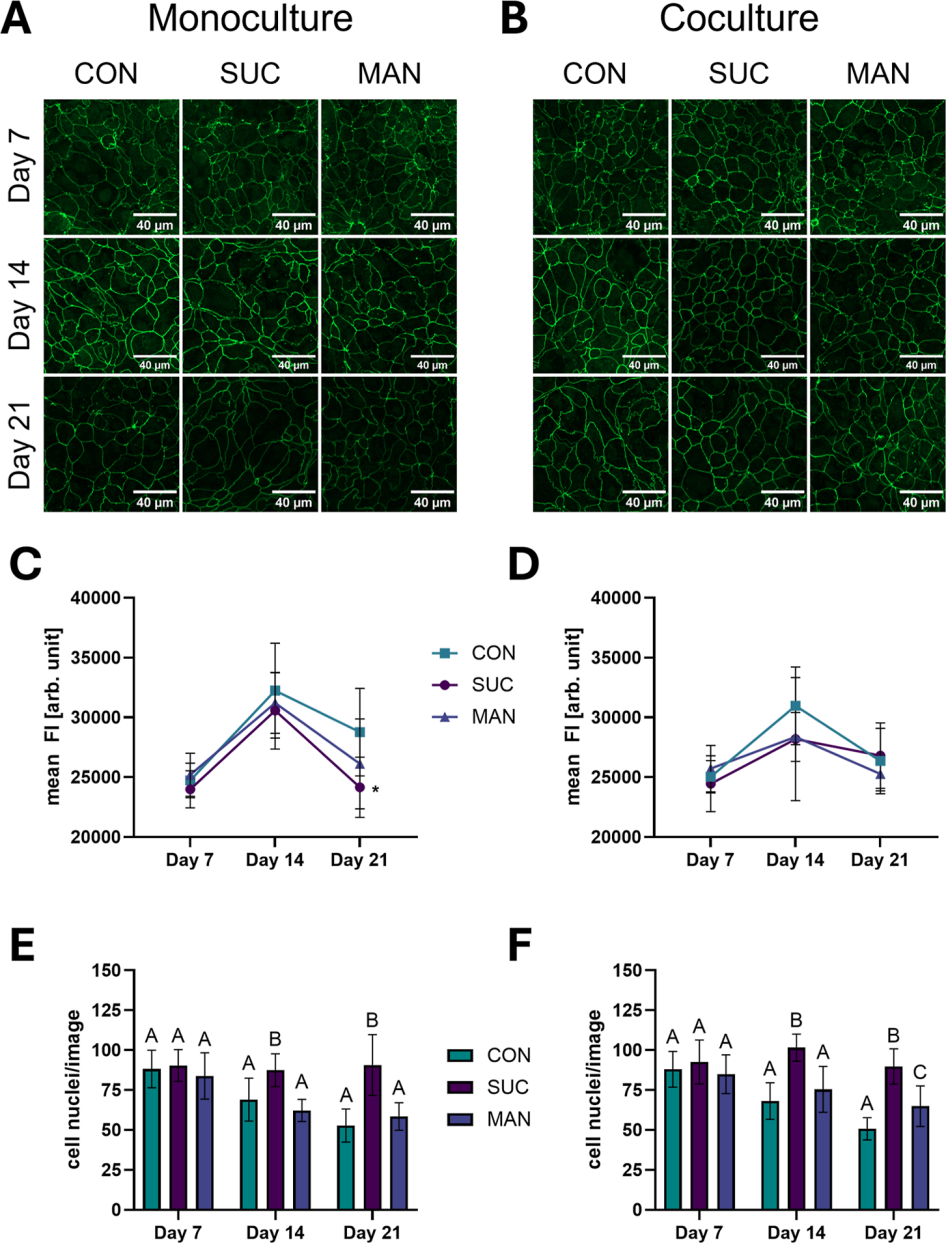
Immunostaining of TJP1 and quantitative data (presented as mean ± SD with *n* = 3, tr = 3–5) of the monoculture (A,C) and coculture (B,D) of the control (CON), treatment with 150 mM sucrose (SUC) or 150 mM mannitol (MAN). Number of cell nuclei per image are presented as mean ± SD (*n* = 3, tr = 3–5) for the monoculture (E) and coculture (F). Significant difference (One-Way ANOVA with Dunnett’s multiple comparisons test) to the corresponding control is indicated with *p < 0.05. Significant differences (*p* < 0.05) between treatments and control (One-Way ANOVA with Tukey’s multiple comparisons test) are indicated by different letters (A, B, and C).

To estimate the differences in the cell size following the long-term treatment, we counted cell nuclei per image. Overall, the number of nuclei per image was similar in the monoculture and the coculture models (Figure 6E,F). The number of nuclei per image decreased over time in the control samples and after 150 mM mannitol treatment, which argues for an increase in the cell size over time. In contrast, the number of cell nuclei remained at a constant level after sucrose treatment over a test period of 21 days, indicating a proliferative effect of sucrose. A significantly different impact of the sucrose treatment on the cell number per image, compared to the control and mannitol treatment, was found for days 14 and 21 for both models. The two-way ANOVA revealed a significant impact of the treatment, cultivation time, and a combination of both for the two models.

## 4 Discussion

The aim of this study was to investigate the impact of a long-term treatment of sucrose and noncaloric sweeteners on markers of intestinal permeability in two models of the intestinal barrier function.

First, the permeability of lucifer yellow after treatment with sucrose or noncaloric sweeteners during the differentiation of the Caco-2 models was measured to investigate the barrier function. Previous studies showed that administration of 30% sucrose solution to mice for 8 weeks resulted in an increased permeability of FITC-Dextran (4 kDa) and cell survival of intestinal epithelial cells.^23^ Similarly, in the present study, the permeability was increased after treatment with 150 mM sucrose in the monoculture at all time points. However, treatment with equi-molar and equi-sweet concentrations of sucralose, NHDC, and rebaudioside M had no impact. In contrast to our findings, Shil et al.^8^ reported an increased permeability of undifferentiated Caco-2 cells after treatment with the noncaloric sweeteners sucralose and aspartame in a concentration of 0.1 mM for 24 h via a pathway involving the sweet taste receptor subunit TAS1R3. These contrary findings may be due to the different degrees of differentiation that go along with TJ formation. Another possibility is that the permeability increases following the treatment, but due to habituation, the permeability reaches control level again after a couple of days. Shorter time intervals between the measuring points are necessary to confirm this theory.

The coculture model was more resistant to the treatments, suggesting a protective effect of the mucus layer produced by HT29-MTX-E12 cells. A beneficial protective effect of mucins for the intestinal barrier function has been also described by Li et al.^24^ in a previous in vitro study and might also provide an explanation for the discrepancy of our results to the results reported by Shil et al.,^8^ who used undifferentiated Caco-2 cells in their study. Although both are cell culture models, the coculture model is generally thought to be able to map the intestinal barrier function in a more physiological way.^12^ In this study, an increased mucus abundance and a decreased TEER value of the coculture model, compared to the Caco-2 monoculture, were achieved through the addition of mucus-producing goblet cells (HT29-MTX-E12). As the caloric and noncaloric sweeteners used in the present study did not increase permeability in a similar manner when used in an equi-sweet concentration, we conclude that the sweetness of the compounds is not the cause for the increased permeability, but the results suggest a substance-specific effect of sucrose. We next aimed to explore the mechanism of sucrose on the permeability. To evaluate whether the sweet taste receptor subunit TAS1R3 plays a role in the increased permeability after treatment with 150 mM sucrose, the TAS1R3-inhibitor lactisole was used in combination with 150 mM sucrose. We first confirmed that the used Caco-2 cells express the encoding gene for TAS1R3 (Figure S3) and that the administration of the inhibitor in comparison to the media control did not lead to a decrease in the permeability of LY (Figure S6). This addition did not decrease the permeability of LY in comparison to the sucrose treatment alone in the monoculture or the coculture, excluding TAS1R3 as the driving force.

As sucrose only increased the permeability in higher concentrations, osmotic stress might be a cause for the effect as hyperosmotic conditions were previously shown to affect the barrier function of Caco-2 cells.^25^ Thus, we used a treatment with mannitol in the same concentration as sucrose to investigate a potential osmotic effect of the sucrose treatment. The monoculture and the coculture models reacted differently to the mannitol treatment. While the permeability of LY after osmotic stress with 150 mM mannitol stayed at the level of control in the coculture model, the permeability of the monoculture model increased with time. More specifically, the permeability of the monoculture after mannitol treatment was lower compared to the control on day 7 and developed into a moderately increased permeability over time. The initial decrease after mannitol treatment on day 7 suggests a defense mechanism to fight the osmotic stress. However, this trend is contrary to the effect after sucrose treatment, where the effect decreased with time, which argues for a habituation effect. However, the treatments caused a paracellular permeability of >3%, indicating a damaged monolayer.^26^ A robust two-way ANOVA revealed that the cultivation time, the model, and the combination of both have a significant impact on the permeability. These results suggest that (i) the coculture is more resistant to the osmotic stress and (ii) the osmotic stress is not the main driving force behind the effect caused by 150 mM sucrose.

As it has been described that glucose can increase the paracellular permeability of Caco-2 cells by activating SGLT1 and subsequently myosin light chain (MLC) phosphorylation, we next investigated a pathway involving glucose transporters.^3^ It has been reported that Caco-2 cells express sucrase-isomaltase,^27,28^ an enzyme that cleaves sucrose into glucose and fructose. This suggests that the effect of sucrose might be due to a similar pathway involving SGLT1 and MLCK. First, we analyzed the changes in the gene expression of the genes encoding SGLT1 (*SLC5A1*) and MLCK (*MYLK*) after treatment with sucrose in comparison to that of mannitol. Compared to the control, treatment with sucrose decreased the expression of *SLC5A1*, while treatment with mannitol increased the expression over time. These results suggest a counter regulation to decrease the SGLT1 abundance and therefore a reduced activation of MLCK, which could be a mechanism to adapt to the increased permeability caused by sucrose, by counter regulating the signaling cascade for the opening of the tight junctions via SGLT1 and MLCK activation.^1,3,6^

Interestingly, the gene expression of *MYLK* was upregulated in both models and treatments at all time points, supporting a pathway via SGLT1 and MLCK. However, a confirmation on a functional level is lacking as the use of inhibitors for SGLT1 (phloridzin^3^) and MLCK (PIK^7^) and incubation with 150 mM glucose or 150 mM sucrose for the whole time course of 21 days led to low viabilities, therefore not suitable for the experimental setup.

MLCK plays an important role in the modulation of the paracellular permeability. Activation of this enzyme leads to phosphorylation of the MLC, which results in the contraction of the actomyosin ring and opening of the TJs. Consequently, this leads to an increased paracellular permeability.^1^

To investigate the impact of the long-term treatment of sucrose and the osmotic control mannitol on TJs, the gene expression of selected TJs was analyzed. Overall, a significant upregulation after sucrose and mannitol treatment for the TJ genes *TJP1*, *OCLN*, *CLDN1*, *CLDN4*, and *CLDN7* in both models was shown. As these are important for the sealing of the paracellular pathway, this upregulation indicates a regulation to counteract the increased permeability. This fast response is in line with findings of Grauso et al.,^25^ who stated that the expression of TJ genes was quickly upregulated after osmotic pressure and increased permeability. In contrast, the gene expression of the pore forming claudin-2 was significantly downregulated after sucrose treatment in both models at all time points. Pores formed by claudin-2 are important for the flux of water and ions such as sodium through the paracellular space. The removal of claudin-2 from MDCK cells reduced the permeation of sodium ions,^29^ while the addition reduced the barrier function in MDCK I cells.^2^ The loss of claudin-4 and claudin-7 expression showed enhanced sodium ion permeability in MDCK cells, indicating their function as paracellular barrier to sodium ions.^29^ These results support the idea that the decreased claudin-2 and increased claudin-4 and claudin-7 gene expressions are counter-regulations to reduce the sodium ion flux and therefore reduce SGLT1-mediated permeability and glucose uptake.

To assess the distribution of TJP1, immunostaining was conducted. According to the assumption of a counter-regulation on the gene expression level, we found that the high overexpression of the *TJP1* gene was not in line with the protein abundance after sucrose and mannitol treatment. Although protein abundance measurements by immunofluorescence is considered semiquantitative, it can be used to assess the absolute protein concentration after optimization.^30^ However, it is important to point out that the results for the protein abundance of TJP1 in this study are based on image analysis of the presented TJP1 immunostaining and may deviate from protein expression experiments such as Western blot or ELISA. However, we did not observe differences in the distribution of TJP1 in the control and treatments concerning the shape of the cells, which supports findings from Grauso et al.,^25^ that cell morphology was similar to the control of Caco-2 cells after osmotic pressure. However, in the present study, the number of cell nuclei per image was higher after sucrose treatment than the control and mannitol treatments on days 14 and 21 in both cultures. These findings are in line with findings from an in vivo study,^23^ where mice were fed with 30% sucrose for 8 weeks. The authors found an increase in villi length in the proximal and distal small intestine, concluding that sucrose enhanced cell survival.

As a main limitation of the study, it is important to consider that results from in vitro studies cannot be transferred directly to in vivo situations. In addition, the cell model was too sensitive to be treated for the whole time span with certain inhibitors, which prevented additional evidence that sucrose increases intestinal permeability via SGLT1 and MLCK.

To the best of our knowledge, this is the first study that investigated the effect of a long-term treatment with caloric and noncaloric sweeteners in equi-sweet concentrations on two models for the intestinal barrier function.

In conclusion, only sucrose but not the tested noncaloric sweeteners increased the permeability of LY after a long-term incubation in the monoculture. The coculture was more resistant to the treatments and osmotic stress, suggesting a protective effect of the mucus layer.

The osmotic stress and sweet taste receptor subunit TAS1R3 were excluded as the driving force for the increased permeability after sucrose treatment. Instead, gene expression analysis of *SLC5A1* and TJ genes argue for a counter-regulation and the importance of SGLT1 for increased permeability. This is further supported by the decreased protein abundance of TJP1 and the high upregulation of the gene encoding TJP1 after sucrose and mannitol treatment. In addition to the increased permeability, sucrose enhanced the cell survival, leading to a higher cell density. However, more research is needed to elucidate the mechanisms behind the increased permeability with long-term treatments of sucrose.

Our results point to the importance of the mucus layer, which can contribute to more correctly assessing the effect of sweeteners on the intestinal barrier and support the use of coculture models for future studies.

## Supplementary Material



## Notes

TEER and lucifer yellow permeability; Alcian blue staining; gene expression of TAS1R3, SLC5A1, and SLC2A2 over time; neutral red uptake; TEER of Caco-2 monoculture and coculture with HT29-MTX-E12 cells; and lucifer yellow permeability after treatment with lactisole (PDF)

## Abbreviations

BSA: bovine serum albumin
CLDN1: claudin 1
CLDN2: claudin 2
CLDN4: claudin 4
CLDN7: claudin 7
DMSO: dimethyl sulfoxide
GAPDH: glyceraldehyde-3-phosphate dehydrogenase
GLUT2: glucose transporter 2
HBSS: Hanks’ balanced salt solution
HEPES: 4-(2-hydroxyethyl)-1-piperazineethanesulfonic acid
HPRT1: hypoxanthine phosphoribosyltransferase 1
LAC: lactisole
LY: lucifer yellow
MAN: mannitol
MDCK: Madin–Darby canine kidney
MLC: myosin light chain
MLCK: myosin light chain kinase
MYLK: myosin light chain kinase (gene)
*n*: biological replicates
NHDC: neohesperidin dihydrochalcone
OCLN: occludin
PBS: phosphate-buffered saline
PIK: membrane permeant inhibitor of MLCK
qPCR: quantitative real time PCR
REBM: rebaudioside M
SGLT1: sodium glucose cotransporter 1
SLC2A2: glucose transporter 2 (gene)
SLC5A1: sodium glucose cotransporter 1 (gene)
SUC: sucrose
SUCRA: sucralose
TAS1R3: taste 1 receptor member 3
TEER: transepithelial electrical resistance
TJ: tight junction
TJP1: tight junction protein 1
tr: technical replicates
