# Supplementary material for: Long-Term Exposure to Sucrose during the Differentiation Process Increases Permeability Independently of TAS1R3 in In Vitro Models of the Intestinal Barrier Function
Source: J Agric Food Chem. 2025 Jul 16;73(30):18763–73. doi: 10.1021/acs.jafc.5c02797 (PMC12314896; doi:10.1021/acs.jafc.5c02797)
Supplement: Supplementary file 1 [file jf5c02797_si_001.pdf]

Supporting Information

Long-term exposure to sucrose during the differentiation process increases permeability independently of TAS1R3 in in vitro models of the intestinal barrier function

Markus L. Rechl [1,2,3], Evelin Balika [1,3], Sascha Oberle [1,3], Verena Preinfalk [1,2,3], Sarah Stadlmayr [1,3], Jana Rasztovits [1,3], Jakob P. Ley [4], Barbara Lieder [1, 3, 5] *

* E-Mail: barbara.lieder@univie.ac.at, Barbara.lieder@uni-hohenheim.de (BL)

[1] Christian Doppler Laboratory for Taste Research, Faculty of Chemistry, University of Vienna, Josef-Holaubek-Platz 2, Vienna, 1090, Austria

[2] Vienna Doctoral School in Chemistry (DoSChem), Währinger Straße 42, Vienna, 1090, Austria

[3] Institute of Physiological Chemistry, Faculty of Chemistry, University of Vienna, Josef-Holaubek-Platz 2, Vienna, 1090, Austria

[4] Symrise AG, Muehlenfeldstrasse 1, Holzminden, 37603, Germany

[5] Institute of Clinical Nutrition, Department of Human Nutrition and Dietetics, University of Hohenheim, Fruwirthstr. 12, Stuttgart, 70593, Germany

Content of supporting information

TEER and LY of Caco-2, HT29-MTX-E12 and coculture (**Figure S1**)..................2

Alcian blue staining protocol and images (**Figure S2**)..3

Gene expression analysis of TAS1R3 and SLC2A2 (**Table S1** and **Figure S3**) ...4

Neutral red uptake assay (**Figure S4**) 5

TEER values before LY permeability assay (**Figure S5**).......................................6

LY permeability assay after treatment with 1 mM lactisole (**Figure S6**) 7

References...7

Characterization of the cell culture models

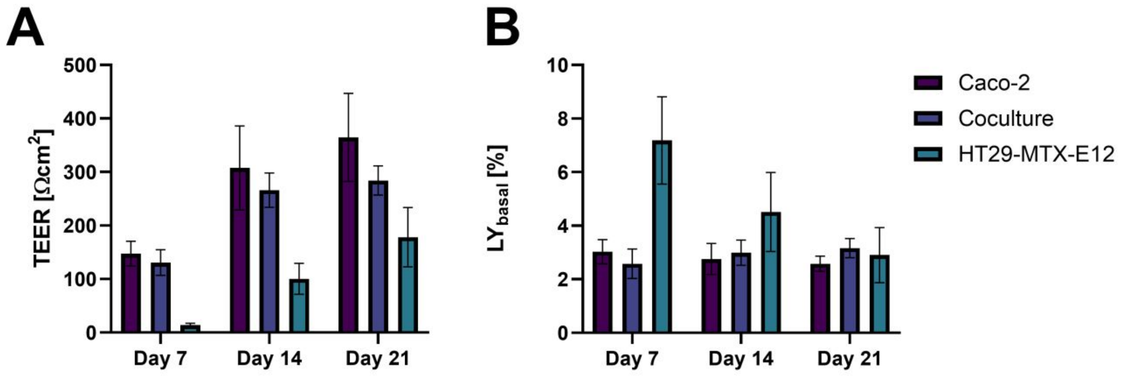

Figure S1. (A) Transepithelial electrical resistance (TEER) and (B) paracellular permeability of lucifer yellow (LY) after 60 minutes of the Caco-2 monoculture, HT29-MTX-E12 monoculture and 9+1 coculture on day 7, 14 and 21 depicted as mean ± SD (n = 3-10).

Mucus staining with alcian blue

For the assessment of mucus production, an alcian blue staining was conducted, according to Pan et al [1]. Therefore, Caco-2 cells, HT29-MTX-E12 cells and the coculture of these two cell lines in a ratio of 9+1 respectively, were seeded on glass cover slips in 24-well plates with a density of 3.64 x 10^5 cells per well. The growth medium was changed every 2-3 days and cells were cultivated until day 14 or 21 after seeding. On day 14 or day 21, cells were washed with 500 µL PBS and fixed with 4% formaldehyde (Sigma Aldrich, Vienna, Austria) for 30 minutes at room temperature. After two washing steps with PBS, 500 µL of 0.1% hydrochloric acid (VWR, Vienna, Austria) was added to reduce the pH. Mucus was stained by addition of 500 µL 1% alcian blue solution (Alcian Blue 8 GS, Carl Roth, Karlsruhe, Germany) in 3% acetic acid (Carl Roth, Karlsruhe, Germany) with pH = 2.5, for 30 minutes at room temperature. Stained cells were washed twice with PBS and images were taken, using a transmitted light microscope (IT400 Trino, VWR, Vienna, Austria) and a 12 MP dual camera.

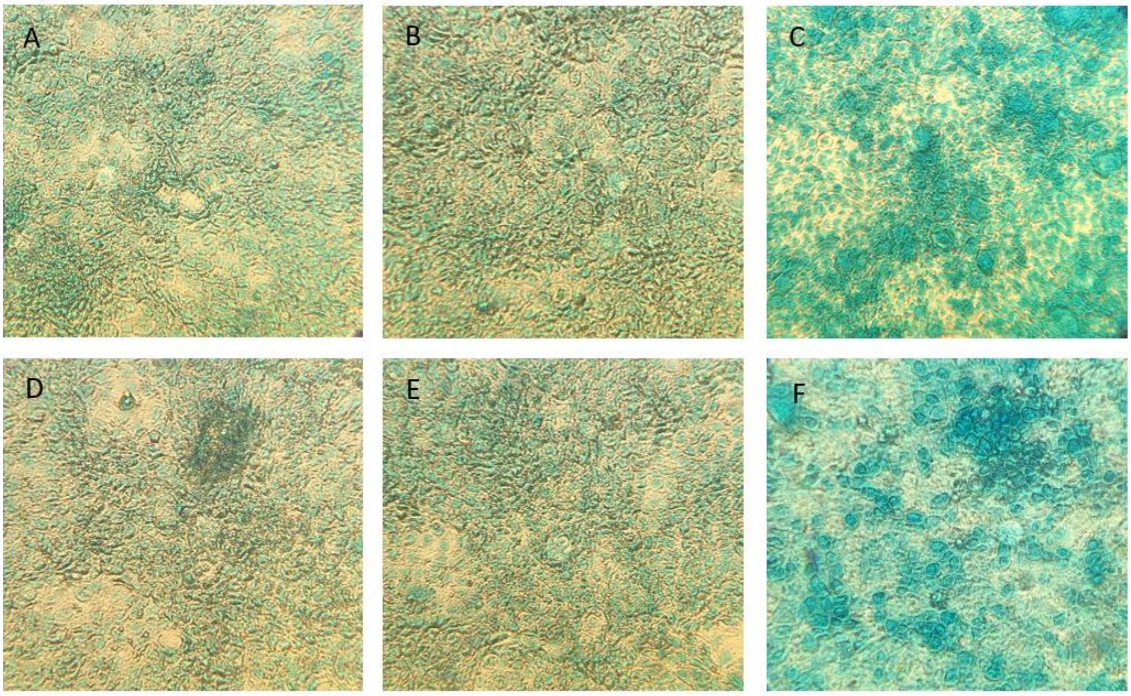

Figure S2. Alcian blue staining of (A) Caco-2 monoculture on day 14, (B) coculture (9+1) of Caco-2 and HT29-MTX-E12 cells on day 14, (C) HT29-MTX-E12 monoculture on day 14, (D) Caco-2 on day 21, (E) coculture on day 21 and (F) HT29-MTX-E12 on day 21.

Gene expression analysis of *TAS1R3* and *SLC2A2*

Table S1. Primer sequence for *TAS1R3* and *SLC2A2*.

Gene	Sequence 5’-3’	C_{Final} [nM]	Source
TAS1R3	FW: CCGCAGTGTGACTGCATCAC<br>RV: CACGCTATACACAGCTGCGTAGA	300	Primer Blast
SLC2A2	FW: CATGCTCTGGTCCCTGTCTGTATC<br>RV: AACCCCATCAAGAGAGCTCCAACT	100	[2]

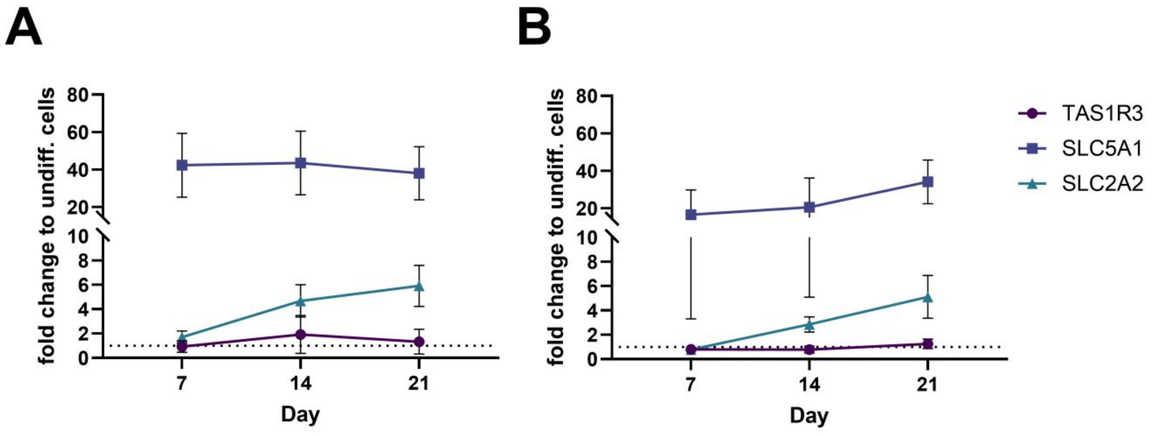

Figure S3. Gene expression of *TAS1R3*, *SLC5A1*, *SLC2A2* of (A) the Caco-2 monoculture and (B) the coculture on day 7, 14 and 21, presented as fold change to undifferentiated cells (day 0) after normalization to the reference genes *HPRT1* and *GAPDH* as mean ± SD (n=3-4, tr=2-3).

Neutral red uptake assay on day 21

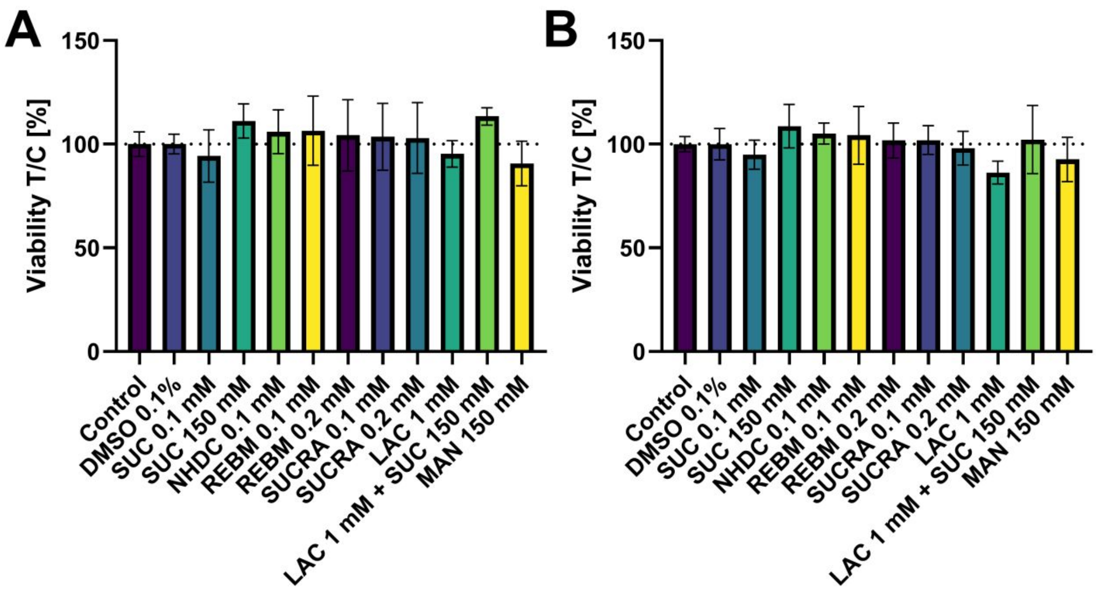

Figure S4. Neutral red uptake relative to the corresponding control [%] of the (A) monoculture and (B) coculture on day 21 after treatment, depicted as mean ± SD (n=3-7, tr=2-3). Abbreviations of the treatments: DMSO (dimethyl sulfoxide), NHDC (neohesperidin dihydrochalcone), SUC (sucrose), REBM (rebaudioside M), SUCRA (sucralose), MAN (mannitol) and LAC (lactisole).

TEER values before lucifer yellow permeability assay

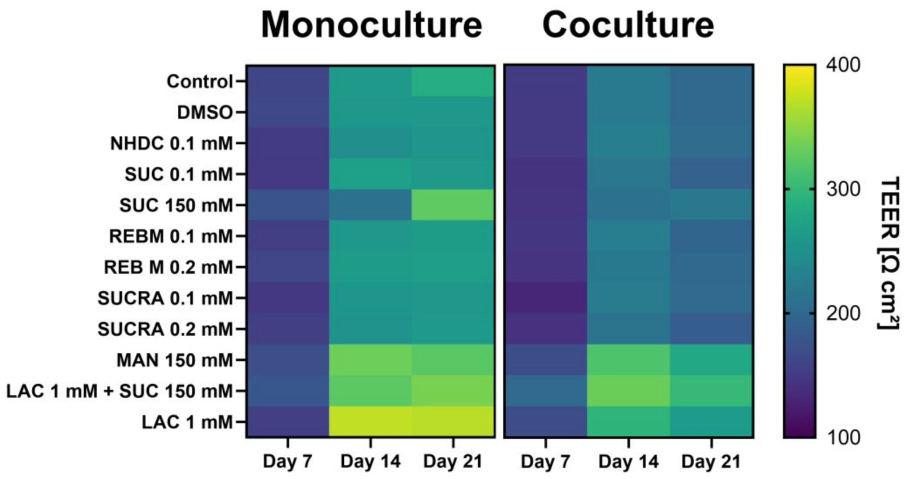

Figure S5. Mean (n=3-5) transepithelial electrical resistance (TEER) [Ω cm²] of the Caco-2 monoculture and the coculture of Caco-2 cells and HT29-MTX-E12 cells on day 7, 14 and 21 before lucifer yellow permeability assay. Abbreviations of the treatments: DMSO (dimethyl sulfoxide), NHDC (neohesperidin dihydrochalcone), SUC (sucrose), REBM (rebaudioside M), SUCRA (sucralose), MAN (mannitol) and LAC (lactisole).

Lucifer yellow after treatment with 1 mM lactisole

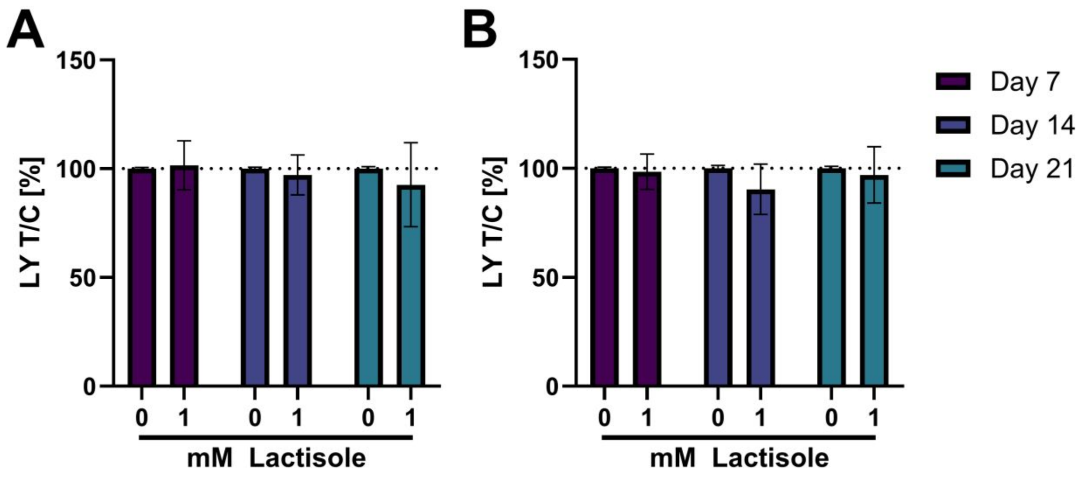

Figure S6. Paracellular permeability of lucifer yellow (LY) relative to the control [%] of (A) the monoculture and (B) coculture on day 7, 14 and 21 depicted as mean ± SD (n=4, tr=2) after treatment with 1 mM lactisole (LAC). Mann Whitney test showed no significant difference ($p < 0.05$) between the treatment and the corresponding control.

References

(1) Pan, F.; Han, L.; Zhang, Y.; Yu, Y.; Liu, J. Optimization of Caco-2 and HT29 co-culture in vitro cell models for permeability studies. *Int J Food Sci Nutr* **2015**, *66* (6), 680-685. DOI: 10.3109/09637486.2015.1077792.

(2) Preinfalk, V.; Kimmeswenger, I.; Somoza, V.; Lieder, B. Dipeptidyl-peptidase 4 (DPP4) mediates fatty acid uptake inhibition by glucose via TAS1R3 and GLUT-2 in Caco-2 enterocytes. *Heliyon* **2024**, *10* (9), e30329. DOI: 10.1016/j.heliyon.2024.e30329.
